# Genomic signature of highland adaptation in fish: a case study in Tibetan Schizothoracinae species

**DOI:** 10.1186/s12864-017-4352-8

**Published:** 2017-12-06

**Authors:** Chao Tong, Fei Tian, Kai Zhao

**Affiliations:** 1 Key Laboratory of Adaptation and Evolution of Plateau Biota, Qinghai Key Laboratory of Animal Ecological Genomics, Laboratory of Plateau Fish Evolutionary and Functional Genomics, Northwest Institute of Plateau Biology, Chinese Academy of Sciences, Xining, 810001 China; 2 University of Chinese Academy of Sciences, Beijing, 100049 China; 3 Department of Biology, University of Pennsylvania, Philadelphia, PA 19104-6018 USA

**Keywords:** Comparative genomics, Schizothoracinae, Highland adaptation, Positive selection, Innate immunity

## Abstract

**Background:**

Genome-wide studies on highland adaptation mechanism in terrestrial animal have been widely reported with few available for aquatic animals. Tibetan Schizothoracinae species are ideal model systems to study speciation and adaptation of fish. The Schizothoracine fish, *Gymnocypris przewalskii ganzihonensis* had underwent the ecological niche shift from salt water to freshwater, and also experienced a recent split from *Gymnocypris przewalskii przewalskii*. In addition, *G. p. ganzihonensis* inhabited harsh aquatic environment including low temperature and hypoxia as well as other Schizothoracinae species, its genetic mechanism of highland adaptation have yet to be determined.

**Results:**

Our study used comparative genomic analysis based on the transcriptomic data of *G. p. ganzihonensis* and other four fish genome datasets to investigate the genetic basis of highland adaptation in Schizothoracine fish. We found that Schizothoracine fish lineage on the terminal branch had an elevated dN/dS ratio than its ancestral branch. A total of 202 gene ontology (GO) categories involved into transport, energy metabolism and immune response had accelerated evolutionary rates than zebrafish. Interestingly, we also identified 162 genes showing signature of positive selection (PSG) involved into energy metabolism, transport and immune response in *G. p. ganzihonesis*. While, we failed to find any PSG related to hypoxia response as previous studies.

**Conclusions:**

Comparative genomic analysis based on *G. p. ganzihonensis* transcriptome data revealed significant genomic signature of accelerated evolution ongoing within Tibetan Schizothoracinae species lineage. Molecular evolution analysis suggested that genes involved in energy metabolism, transport and immune response functions in Schizothoracine fish underwent positive selection, especially in innate immunity including toll-like receptor signaling pathway genes. Taken together, our result as a case study in Schizothoracinae species provides novel insights in understanding the aquatic animal adaptation to extreme environment on the Tibetan Plateau, and also provides valuable genomic resource for further functional verification studies.

**Electronic supplementary material:**

The online version of this article (10.1186/s12864-017-4352-8) contains supplementary material, which is available to authorized users.

## Background

It is of evolutionary interest to understand that how wildlife adapts to high altitude [1]. With an average elevation above 4000 m, the Tibetan Plateau (TP) is one of the earth’s most significant continental-scale highlands [2] imposes an extremely inhospitable environment on most wildlife, including hypoxia, high ultraviolet radiation and low temperatures [3, 4]. Past research had indicated the adaptation of local wildlife to harsh living challenges. Recent studies employing genome-wide approaches on Tibetan terrestrial animal have primarily focused on response to hypoxia and energy metabolic pathways, including yak [4], Tibetan antelope [5], ground tit [2], Tibetan mastiff [6], Tibetan dog [7], Tibetan chicken [8]. Nevertheless, we know little about the mechanism of Tibetan aquatic animal adaptation to aquatic environment on the TP. Specifically, genetic mechanisms of adaptation in Schizothoracine fish have yet to be determined. Therefore, it may provide novel insights for understanding the mechanism of highland adaptation of Tibetan wildlife.

**Fig. 1:**
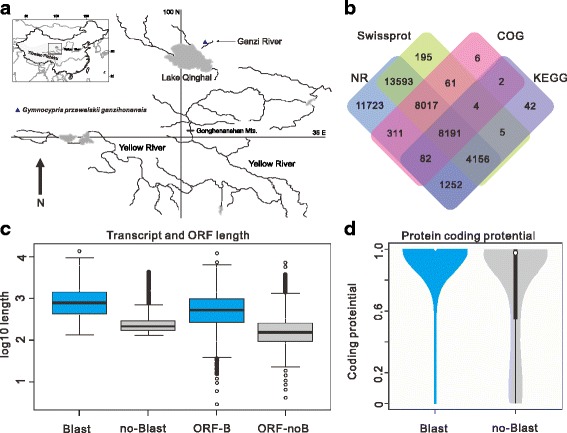
Sampling site and annotation of *G. p. ganzihonensis* transcriptome. **a** The sampling map was created using the ArcGIS v10.1 (ESRI, CA, USA) and Adobe Illustrator CS5 (Adobe Systems Inc., San Francisco, CA). **b** Venn diagram shows shared and distinct genes under the annotations of NR, Swiss-Prot, COG and KEGG databases. Numbers indicating how many unigenes were annotated by each database. **c** Boxplot shows sequence characterization of the transcripts with and without detected homologs. **d** Protein coding potential were determined by CPAT and illustrated by boxplot

The Schizothoracinae is the largest and most diverse taxon of the TP ichthyofauna, which are distributed throughout the TP and its peripheral regions [9, 10]. Past research had revealed that Schizothoracinae species had well adapted to the harsh aquatic environment on the TP, including hypoxia, low temperature and even high salinity [9, 11–13], making them excellent models for investigating the genetic mechanism of aquatic animal adaptation to the extreme environment at high altitude. In addition, increasing studies focused on the speciation mechanism of the Schizothoracine fish and the uplift of the Tibetan Plateau [14–17]. A Schizothoracine fish, *Gymnocypris przewalskii ganzihonensis* is the only fish inhabiting the Ganzi River (Fig. 1a). Another Schizothoracine fish, *Gymnocypris przewalskii przewalskii* is also the only fish inhabiting the Lake Qinghai (the largest salt lake in China). Previous research had indicated that the Ganzi River once flowed into the Lake Qinghai before the Wei-Jin-Nanbei Dynasty (200 to 589 A.D) [18]. An additional survey had revealed that the Ganzi River had disconnected to the Lake Qinghai as offshore great sand dune movement and shrinking of lake shoreline, which resulted in *G. P. przewalskii* colonized the freshwater habitat [18]. In addition, the taxonomists named this fish species as *G. p. ganzihonensis* based on morphological data [9], and also have been supported by mitochondrial evidences [17, 19–21]. Obviously, *G. p. ganzihonensis* had undergone the transition from salt water to freshwater, and this species also faced the challenges due to the low temperature and hypoxia environment in accord with other Schizothoracinae species. Therefore, it is an interesting issue to investigate the highland adaptation in fish species using *G. p. ganzihonesis* as a case study in Schizothoracinae.

Recent advances in sequencing technologies have offered the opportunity to map and quantify transcriptome in almost any species of interest that do not currently have a reference genome [22]. Noteworthy, most Schizothoracinae species are polyploidy, tetraploid, and even sixteen-ploid [9]. Transcriptome sequencing technology have been successfully applied in many polyploidy cases [23, 24], which is a rapid and effective approach to obtain massive protein-coding genes and molecular markers. This technology could facilitate investigations into the genetic basis of adaptations. Here we sequenced and generated the transcriptome of *G. p. ganzihonensis* as a case study in Tibetan Schizothoracinae species. We then performed comparative genomic analysis together with other previously available fish genomes to reveal the potential genetic mechanism of highland adaptation in fish.

## Methods

### Sample collection

Ten adult *G. p. ganzihonensis* samples were captured and identified from the Ganzi River using gill nets. Gender was determined for each specimen. Next, four individuals (2 male and 2 female) were selected and dissected after anesthesia with MS-222 (Solarbio, Beijing, China). Gill and kidney from each individual were collected respectively, and then immediately stored in liquid nitrogen at −80 °C.

### RNA extraction and transcriptome sequencing

Total RNAs (*n* = 8, 4 gills and 4 kidney) of two tissues were isolated from each of four individuals using TRIzol (Invitrogen, Carlsbad, CA) according to the manufacturer’s instructions. The quantity and quality of total RNA was verified by an Agilent 2100 bioanalyzer (Agilent Technologies, Palo Alto, CA) and gel electrophoresis. Approximately 10 μg of each RNA of same tissue from different individual were pooled for transcriptome library preparation (totally two independent libraries, gill and kidney), and sequenced on an Illumina HiSeq™ 2000 platform (parameters: 101-bp paired-end reads, 1 lane).

### Transcriptome assembly and annotation

RNA-seq raw reads from each library were preprocessed to filter residual adapter sequences and low-quality reads (Q < 20), Then all clean reads were assembled using the Trinity v2.2.0 program (https://github.com/trinityrnaseq/trinityrnaseq/releases) with default parameters. Contigs from each sample’s assembly were clustered by CD-HIT program [25] (percent identity: 80%; word size: 5) to generate a set of non-redundant unigenes, with a minimum overlap length of 200 bp. The assembled unigene sequences were aligned with a Blast-X search (cut-off E-value of 1 × 10^−10^) in public NCBI non-redundant (NR), Swiss-Prot, Cluster of Orthologous Groups (COG) databases and Kyoto Encyclopedia of Genes and Genomes (KEGG) database. Gene ontology (GO) terms were obtained from NR hits using Blast2GO (version_3.2) [26] with default parameters. Next, the Getorf program in EMBOSS (version_6.4.0) [27] was applied to obtain the Open reading frames (ORFs) of *G. p. ganzihonensis* genes. The CPAT tool [28] was used to predict the protein-coding potential for the assembled unigenes, with previously downloaded zebrafish dataset (Zv9/danRer7) as the assembly database and 0.38 as the coding probability cutoff.

### Orthologs identification

Orthologs between Schizothoracine fish (*G. p. ganzihonensis*) and zebrafish were identified using reciprocal BLAST best-hit method with an E value cutoff of 1 × 10^−10^ as used in prior investigations [29]. Then 1:1 orthologs between four fish genomes, including zebrafish (*Danio rerio*), fugu (*Takifugu rubripes*), medaka (*Oryzias latipes*), and spotted gar (*Lepisosteus oculatus*) were obtained from Ensembl server using BioMART (JCI_4.2.75) [30]. Noteworthy, only the longest transcript was considered if one gene had multiple transcripts. Each orthologous gene set was aligned using PRANK [31] (parameters: -f = fasta -F -codon -noxml -notree -nopost) and trimmed using GBlocks [31] (parameters: -t = c -b3 = 1 -b4 = 6 -b5 = n). Then we deleted all gaps and “N” from the alignments to lower the effect of ambiguous bases on the inference of positive selection. After deletion process, trimmed alignments shorter than 150 bp after removing sites with ambiguous data were discarded for subsequent analyses.

### Molecular evolution analyses

The CODEML program in PAML 4.7a [32] with the free-ratio model (parameters: model = 1, NSsites = 0, fix_omega = 0, omega = 1) was run on each ortholog, a concatenation of all alignments of the orthologs, and 1000 concatented alignments constructed from 150 randomly chosen orthologys, according to previous studies [29, 33–35]. The parameters of nonsynonymous (Ka or dN), synonymous (Ks or dS) and especially the substitution rate (ω = Ka/Ks or dN/dS) were used to meansure the lineage-specific evolutionary rates of above fish species. Based on GO term date which downloaded from BioMART (Ensembl, JCI_4.2.75), the orthologs were clustered into different functional GO terms and dN, dS and dN/dS ratio for each term was calculated, respectively. Finally, only GO categories with more than 20 orthologs were considered in this section analysis.

In addition, the CODEML program in PAML 4.7a with the branch-site model [36] (parameters: Null hypothesis: model = 2, NSsites = 2, fix_omega = 1, omega = 1) was used to identify positively selected genes (PSGs) in the Schizothoracine fish lineages, with other lineages being specified as the foreground branch. A LRT was constructed to compare a model that allows sites to be under positive selection (ω > 1) on the foreground branch with the null model in which sites may evolve neutrally (ω = 1) and under purifying selection (ω < 1) with a posterior probability in excess of 0.95 based on the Bayes empirical Bayes (BEB) results [37]. Finally, the *P* values were computed based on rigorous Chi-square statistic adjusted by FDR method and genes with adjusted *P* value <0.05 were treated as candidates under positive selection.

## Results

### Transcriptome sequence analysis and assembly

Two pooled cDNA libraries derived from gill and kidney tissues of Schizothoracine fish, *G. p. ganzihonensis* were prepared and sequenced, totally generated 85,371,306 (gill) and 88,787,918 (kidney) raw 101-bp paired-end (PE) reads, respectively. After trimming adapters and removing low-quality reads, a total of 78,605,558 (gill) and 80,382,460 (kidney) clean reads were obtained from gill and kidney dataset, respectively. Finally, a total of 132,554 unigenes ranged from 201 to 16,310 bp, with an average length of 952 bp and an N50 of 1836 bp (Additional file 1: Table S1), the length distribution of all transcripts is shown in Additional file 2: Figure S1.

### Functional annotation

To comprehensively annotate the transcriptome of *G. p. ganzihonensis*, all unigenes were queried against several public databases. A total of 94,321 (71.15%) sequences yielded at least one significant match to an existing gene model in Blast-X search (Fig. 1b, Additional file 3: Table S2). Statistics results of COG and GO classification of all annotated unigenes were shown in Additional file 4: Figure S2 and Additional file 5: Figure S3. Almost half (47.82%, *n* = 63,404) of homologs aligned to known proteins have identified between 80% and 100%. Due to fact of *G. p. ganzihonensis* is phylogenetically closer to zebrafish than some other fish species with complete genomic resources, it is not surprising that 81.72% (*n* = 51,812) of the best hits were similar with model organism zebrafish (Additional file 6: Table S3). Next, the assembly unigene dataset was divided into two subsets to characterize the sequence features in detail, including unigenes with and without protein homology in NR, namely “Blast” and “no-Blast” respectively. The “Blast” subset had significantly larger unigenes length and longer ORFs than the “no-Blast” subset with *P* value <2.2 × 10^−5^ in Wilcoxon rank sum test (Fig. 1c). In addition, further analysis of the potential for protein coding with CPAT tool showed a significantly lower protein-coding potential in the “no-Blast” subset with *P* value <2.2 × 10^−6^ (Fig. 1d).

### Accelerated evolution of the Schizothoracine fish lineage

**Fig. 2:**
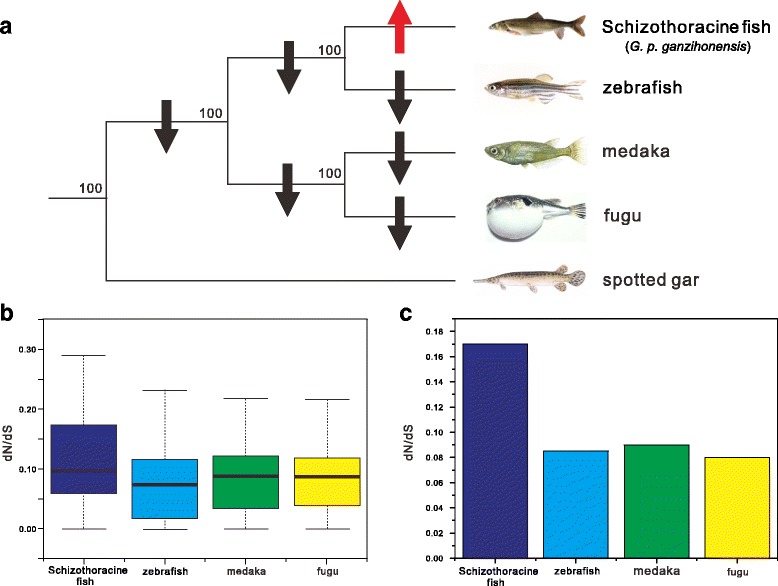
Phylogeny and the dN/dS rate value of Schizothoracine fish and other four fish species. **a** Phylogenetic tree [38] used in present study, spotted gar as outgroup. Red and black arrows indicate decreased or increased terminal dN/dS rate value compared with the ancestral branch. **b** The dN/dS rate value for terminal branches were estimated from each orthologous genes. **c** Average dN/dS rate value of concatenated all orthologs in four fish species

We identified the single-copy orthologs in Schizothoracine fish dataset and zebrafish, fugu, medaka, and spotted gar genome databases, resulting in a total of 6829 orthologs. Next, we used the species tree [38] in conjunction with a branch model constructed in PAML to determine dN, dS, and ω values across all 6829 orthologous genes. The result showed that the averaged ω value was significantly higher than other fish branches with *P* < 2.2 × 10–16 in Wilcoxon rank sum test (Fig. 2a), implied that accelerated function evolution in Schizothoracine fish lineages. In addition, we analyzed the ω value for each branch for a concatenated alignment of all 6829 orthologs and 1000 concatenated alignments constructed from 150 randomly chosen orthologs. Intriguingly, using both comparison strategies, we found that Schizothoracine fish lineage exhibited a significantly higher ω value than other four fish branches in our study (*P* < 2.2 × 10–16) (Fig. 2b and c). Here showed a clear clue was that the Schizothoracine fish branch had an elevated ω value than its ancestral branch and trend to ongoing accelerated evolution under the extreme environment on the TP (Fig. 2a).

**Fig. 3:**
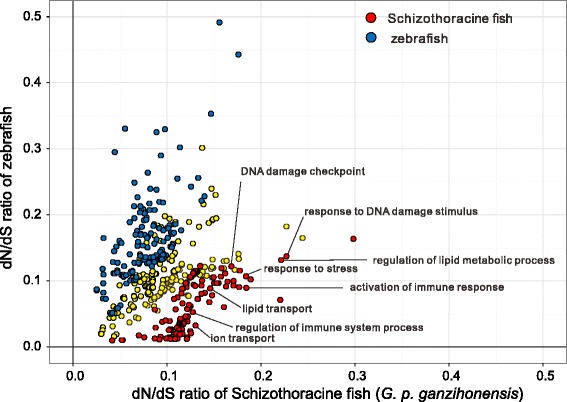
Accelerated evolution within GO category. Scatter plot of mean dN/dS rate value for each GO category in Schizothoracine fish and zebrafish lineages. GO categories with significantly higher mean dN/dS rate value in Schizothoracine fish (red) and zebrafish (blue) are highlighted, respectively. Yellow points represent GO categories with higher but not statistically significant mean dN/dS rate value in both lineages

After a strict filtering analysis, we calculated the mean ω value for each GO category with at least 20 orthologs in Schizothoracine fish and zebrafish lineages, respectively. A total of 202 GO categories showing accelerated evolutionary rate (*P* < 0.05, binomial test) were detected in Schizothoracine fish and 113 in zebrafish (Fig. 3 and Additional file 7: Table S4), which also confirmed overall accelerated evolution in Schizothoracine fish lineage. We then focused on these accelerated categories potentially associated with highland adaptation. Interestingly, these GO categories were mainly involved into four functional groups. One group was mostly related to biotic and abiotic stress, such as “response to DNA damage stimulus” and “activation of immune response”. As the fact is that *G. p. ganzihonensis* in fresh water is split from its ancestor *G. P. przewalskii* in salt water, one groups was related to transport function, such as “ion transport” and “lipid transport”. The other two groups involved in energy metabolism and immune system, such as “regulation of lipid metabolic process” and “regulation of immune system process” (Fig. 3 and Additional file 7: Table S4).

### Candidate genes under positive selection in Schizothoracine fish

**Fig. 4:**
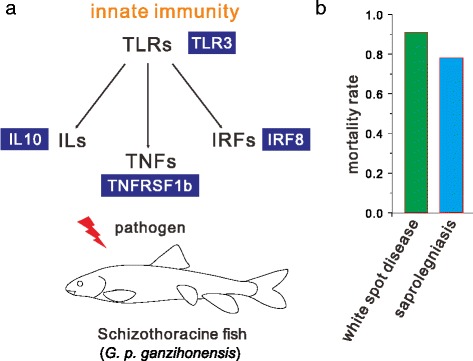
Immune characterizations of Schizothoracine fish. **a** Schematic diagrams of innate immunity and Toll-like receptor (TLR) signing pathway. Four PSGs are highlighted in TLR pathway. The fish innate immune response to pathogens invasion, four major families are involved. TLR = toll-like receptor; IL = interleukin; IRF = interferon regulatory factor; TNF = tumor necrosis factor. **b** The morality rates of two culprit infectious diseases in Schizothoracine fish

To better understand the potential genes contributed to Schizothoracine fish adaptation to TP, we used branch-site model in PAML to identify candidate positively selected genes (PSGs) in Schizothoracine fish lineage. After applying strict filtering criteria, we totally identified 162 PSGs (*P* < 0.05) in *G. p. ganzihonensis* (Additional file 8: Table S5). Intriguingly, the PSGs were also had functions associated with three main groups. The first functional group were related to transport functions, including solute carrier family 12, member 1 (SLC12A1), solute carrier family 7, member 2 (SLC7A2), solute carrier family 38, member 4 (SLC38A4). The PSGs in second group were associated with energy metabolism, including *NADH dehydrogenase 1* (ND1), *ATPase family, AAA domain containing 2* (ATAD2), *ADP-ribosylation factor 3* (ARL3). Innate immunity function group was the third one, such as *toll-like receptor 3* (TLR3), *interferon regulatory factor 8* (IRF8), *interleukin 10* (IL10) and *tumor necrosis factor receptor superfamily, member 1b* (TNFRSF1b (Fig. 4a). Noteworthy, two infectious diseases “white spot” disease and saprolegniasis have been considered as the chief culprits and suffered high mortality rate of Schizothoracine fish when in aquaculture industry rather than native environment (Fig. 4b). This led us to hypothesize whether there is a link between weak immune ability and PSGs in innate immunity. In addition, ten of the candidate PSGs were identified and linked to energy metabolism, including ATP13a, ABCC2a, ATAD2 and MRPL45 (Additional file 8: Table S5). This finding also confirmed that GO category of energy metabolism in *G. p. ganzihonensis* lineage may undergo accelerated evolution.

## Discussion

In evolutionary biology, comparative genomic analysis had been widely applied in understanding the genetic basis of organisms’ speciation [39–41] and adaptation [2, 4, 8, 11, 29, 35]. Although whole genome sequencing data of nonmodel organisms have increasingly become available, most organisms still lack genomic resource. Transcriptome sequencing is an effective and accessible approach to initiate comparative genomic analysis on nonmodel organisms, because it could also contain a large number of protein-coding genes likely enriched for targets of natural selection. In this study, we sequenced and annotated the transcriptome of the Schizothoracine fish, *G. p. ganzihonensis* [9, 18], and identified more than 6000 pairwise orthologs among five fish genomes. Then, we performed comparative genomic analysis on this Schizothoracine fish using its de novo assembly transcriptome dataset and other four fish genomes. Finally, this transcriptome resource could develop our understanding of genetic makeup of highland fishes and provide a foundation for further studies to identify candidate genes underlying adaptation to the Tibetan Plateau of Schizothoracine fishes.

How an organism adapts to environment change is an important issue in evolutionary biology [42]. Adaptive evolution may prefer to proceed at molecular level, expressed by an increase in ratio of nonsynonymous substitutions to synonymous substitutions [43]. Previous studies revealed that terrestrial organisms adapted to life at high altitude by gene family expansion, accelerated evolutionary rate and underwent positive selection on genes associated with specific function [2, 4, 7, 8]. Convergence is an independent evolution of similar physiological or morphological features in different species [44]. Its occurrence could support the hypothesis that specific ecological environment challenges can induce species to evolve in predictable and repeatable ways [45]. Our current analysis results suggested that Schizothoracine fish lineage trends to genome-wide accelerated evolution relative to other fish lineages. Past evidence indicated that accelerated evolution is usually driven by positive selection [32], we therefore speculated that Schizothoracine fish may adaptively speed up its evolutionary rate of genes for better adaptation to extreme environment of the TP. The relaxation of function constraint could possibly trigger accelerated evolution, which the hypothesis should need more cases based on population genomic analyses to support. Furthermore, compared with the ancestral branch, the terminal branch of Schizothoracine fish had underwent an elevated dN/dS ratio, implying that accelerated evolution only in the Schizothoracine fish lineage after diverged from zebrafish (also belonged to Cyprinidae). Previous studies had identified various adaptive processes that may be responsible for highland adaptation in terrestrial animal, including energy metabolism and hypoxia response [2, 4, 46]. Therefore it is not surprising that many GO categories related to energy metabolism and stress response in aquatic animal, Schizothoracine fish. A striking finding of the present study is that “transport function” genes may undergo accelerated evolution, this is consisted of the finding in recent study on the extremely alkaline environment adaptation mechanism in Amur ide, *Leuciscus waleckii* [47]. This finding implied that the adaptive evolution might play important role in this recent split Schizothoracine fish, *G. p. ganzihonesis* in transition of salt water to fresh water.

The functions of candidate PSGs were consistent with above identified functional groups of GO categories exhibiting accelerated evolution. Recent studies revealed the genetic basis of terrestrial animal adaptation to low oxygen and low temperature environment at high altitude [1, 2, 4, 7, 12, 13, 29]. In *G. p. ganzihonesis*, we failed to identify any PSG involved in hypoxia response. This may because the oxygen condition in Tibetan Plateau aquatic environment is different with ground. Previous evidence indicated that abundant and diverse of hydrophyte species in Ganzi River [18], these factors could have positive impacts on the dissolved oxygen content as the plant photosynthesis, which may help to explain the absence of PSGs related to response to hypoxia function. Low temperature is a typical feature of lake and river environment on the TP, which faced up this challenge for all aquatic animals. Accord to previous findings [2, 4], several candidate PSGs involved in energy metabolism were identified. For example, the ATP13a gene that encodes an accessory protein for ATP synthesis and decomposition, suggesting an important role in energy metabolism to adaptation to this low temperature water environment. In addition, SLC family play vital roles in transport function contribute to organism response to dynamic aquatic environment [48]. We also identified several SLC family members shown positive selection in Schizothoracine fish lineage, such as SLC12A1, SLC7A2, SLC38A4. This finding were similar to previous genome-wide study on the Amur ide in extremely alkaline environment [47], which indicated that adaptive evolution within genes involved in transport function contribute to provide novel insight into adaptation to extreme aquatic environment on the Tibetan Plateau. Remarkably, here we provide another novel insight to understand the genetic mechanism of highland adaptation in Schizothoracine fish, the adaptive evolution of innate immunity. Recent evidence showed that Schizothoracine fish is susceptible to infectious diseases and triggered high mortality rate in non-native environment [49–52]. In addition, recent genome-wide study reveal that adaptive evolution of innate immunity contributed to fish well response to pathogen invasion [53]. Intriguingly, we identified significant positive selection signs in TLR3, IRF8, IL10 and TNFRSF1b involved in innate immunity. This finding is similar with our previous reports on the PSGs and neofunctionalization in TLR signaling pathway genes [49, 51, 52], suggesting that adaptive evolution of innate immunity may play important roles in Schizothoracine fish adaptation to high attitude aquatic life.

## Conclusions

In summary, we have sequenced and annotated the first transcriptome of a recent split Schizothoracine fish, *G. p. ganzihonensis*. Comprehensive analyses of over 6000 orthologs among *G. p. ganzihonensis* and four fish genome databases identified evidence for sign of accelerated evolution in *G. p. ganzihonensis* lineage and only the terminal branch of *G. p. ganzihonensis* had an elevated dN/dS ratio than ancestral branches. Number of GO categories involved into energy metabolism, transport and immune response showed rapid evolution in compared with model zebrafish. Intriguingly, we found that many genes showing signature of positive selection in *G. p. ganzihonensis* lineage were enriched in functions associated with energy metabolism, and a novel finding is that PSGs also related to innate immunity, especially in toll-like receptor signaling pathway. While we failed to identify any PSG involved in hypoxia response as previous studies reported in terrestrial animal and several Tibetan fish species. This transcriptome dataset also provides a valuable resource for further functional verification study which will develop our understanding of ecological and evolutionary questions concerning fish species on the TP.

## Notes

Chao Tong and Fei Tian contributed equally to this work.

## Additional files

Additional file 1: Table S1. Summary of sequencing, assembly and analysis of *G. p. ganzihonensis* transcriptome. (DOC 36 kb)

Additional file 2: Figure S1. Length distribution of all transcripts. Transcripts of gill and kidney datasets are calculated respectively. Cumulative length of unigenes is also calculated. (PDF 138 kb)

Additional file 3: Table S2. Annotation of assembled unigenes in *G. p. ganzihonensis* transcriptome. (XLS 41915 kb)

Additional file 4: Figure S2. COG classification of assembled unigenes in *G. p. ganzihonensis* transcriptome. (PDF 22 kb)

Additional file 5: Figure S3. GO classification of assembled unigenes in *G. p. ganzihonensis* transcriptome. (PDF 21 kb)

Additional file 6: Table S3. Species distribution is calculated as a percentage of the total homologous sequences. (XLS 27 kb)

Additional file 7: Table S4. GO categories showing accelerated evolutionary rates within *G. p. ganzihonensis* and *Danio rerio*. (XLS 104 kb)

Additional file 8: Table S5. Positively selected genes (PSGs) identified in *G. p. ganzihonensis*. (XLS 64 kb)

## Abbreviations

COG: Cluster of Orthologous Groups
FDR: false discovery ratio.
GO: Gene ontology
NR: non-redundant
ORFs: Open reading frames
PSG: Positively selected gene
TP: Tibetan Plateau
