# Supplementary material for: Genomic signature of highland adaptation in fish: a case study in Tibetan Schizothoracinae species
Source: BMC Genomics. 2017 Dec 6;18:948. doi: 10.1186/s12864-017-4352-8 (PMC5718033; doi:10.1186/s12864-017-4352-8)
Supplement: Supplementary file 1 — Summary of sequencing, assembly and analysis of G. p. ganzihonensis transcriptome. (DOC 36 kb) [file 12864_2017_4352_MOESM1_ESM.doc]

**Table S1.** Summary of sequencing, assembly and analysis of *G. p. ganzihonensis* transcriptome.

| Dataset name | All | Gill | Kidney |
| --- | --- | --- | --- |
| Total bases (bp) | 14,308,921,620 | 7,074,500,220 | 7,234,421,400 |
| **No. of reads** |  |  |  |
| Raw reads | 174,159,224 | 85,371,306 | 88,787,918 |
| Clean reads | 158,988,018 | 80,382,460 | 78,605,558 |
| Q20^a^ of clean reads | 97.43% | 97.22% | 97.64% |
| **No. of contigs** |  |  |  |
| Total contigs | 479,928 | 239,166 | 240,762 |
| Average contig read length (bp) | 312 | 316 | 307 |
| **No. of unigenes** |  |  |  |
| Total unigenes | 158,087 | 132,554 | 130,604 |
| Distinct clusters | 63,661 | 40,452 | 38,815 |
| Distinct singletons | 94,426 | 92,102 | 91,789 |
| N50^b^ of unigenes (bp) | 1,836 | 1,490 | 1,399 |
| Average unigene read length (bp) | 952 | 736 | 704 |

a Q20: percentage is the proportion of nucleotides with a quality value > 20 in reads.

b N50: unigene length-weighted median.
