# Supplementary figures and images for: Genomic signature of highland adaptation in fish: a case study in Tibetan Schizothoracinae species
Source: BMC Genomics. 2017 Dec 6;18:948. doi: 10.1186/s12864-017-4352-8 (PMC5718033; doi:10.1186/s12864-017-4352-8)

Unigene Length Distribution of *G. p. ganzihonensis*

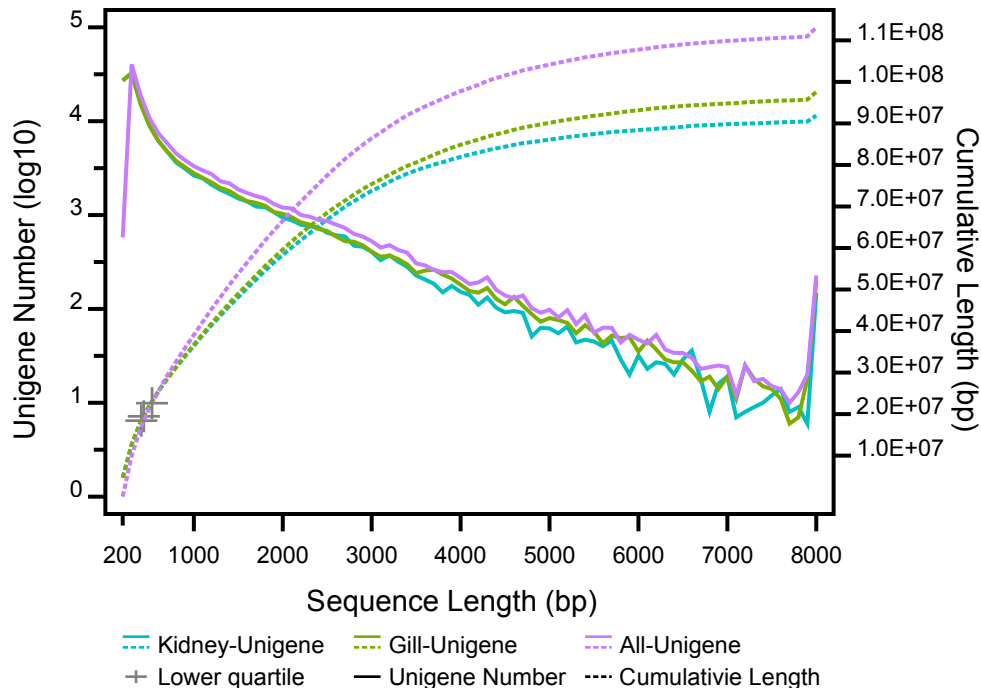

Supplement: Supplementary file 2 — Length distribution of all transcripts. Transcripts of gill and kidney datasets are calculated respectively. Cumulative length of unigenes is also calculated. (PDF 138 kb) [file 12864_2017_4352_MOESM2_ESM.pdf]

COG Function Classification of Tibetan naked carp

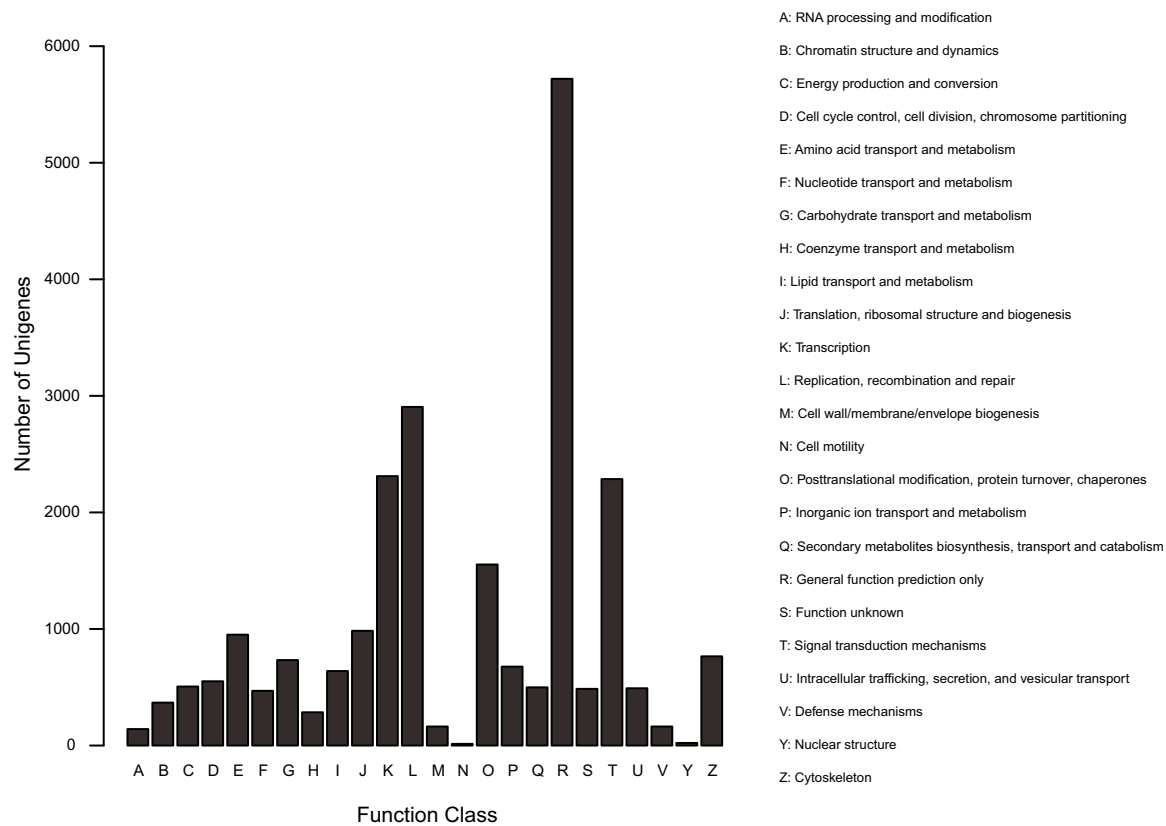

Supplement: Supplementary file 4 — COG classification of assembled unigenes in G. p. ganzihonensis transcriptome. (PDF 22 kb) [file 12864_2017_4352_MOESM4_ESM.pdf]

GO Function Classification of Tibetan naked carp

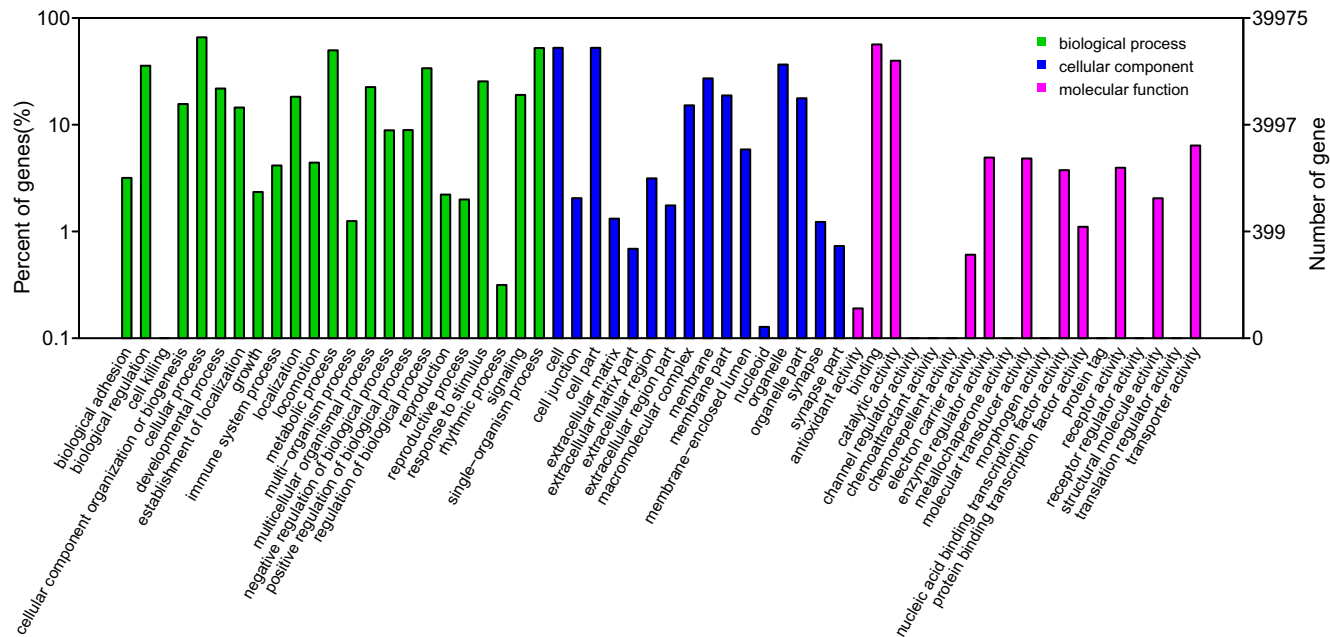

Supplement: Supplementary file 5 — GO classification of assembled unigenes in G. p. ganzihonensis transcriptome. (PDF 21 kb) [file 12864_2017_4352_MOESM5_ESM.pdf]
